# Prevalence of suicidal ideation and associated factors among HIV positive perinatal women on follow-up at Gondar town health institutions, Northwest Ethiopia: a cross-sectional study

**DOI:** 10.1186/s12884-020-03529-z

**Published:** 2021-01-09

**Authors:** Leul Belachew Zewdu, Mebratu Mitiku Reta, Niguse Yigzaw, Koku Sisay Tamirat

**Affiliations:** 1 University of Gondar Comprehensive Specialized Hospital, Gondar, Ethiopia; 2 Department of Internal Medicine, School of Medicine, College of Medicine and Health Sciences, University of Gondar, Gondar, Ethiopia; 3 Department of Psychiatry, School of Medicine, College of Medicine and Health Sciences, University of Gondar, Gondar, Ethiopia; 4 Department of Epidemiology and Biostatistics, Institute of Public Health, College of Medicine and Health Science, University of Gondar, Gondar, Ethiopia

**Keywords:** Suicidal ideation, Perinatal women, Ethiopia

## Abstract

**Background:**

Suicidal behaviors cover a range or continuum of acts from suicidal ideations to a series of actions, commonly known as suicidal attempts or deliberate self-harms. Though different mental disorders related studies were conducted among HIV/AIDS patients, there is a scarcity of information about the magnitude and determinants of suicidal thoughts among perinatal women.

Therefore, this study aimed to determine the prevalence of suicidal ideation and associated factors among HIV positive perinatal women in the study setting.

**Methods:**

An institution-based cross-sectional study was conducted among perinatal women on treatment to the prevention of mother to child transmission of HIV/AIDS at Gondar town health facilities. A total of 422 HIV-positive perinatal women were selected systematically and the data collected through medical record review and interview using a Composite International Diagnostic Interview (CIDI) toolkit. A binary and multivariable logistic regression model was employed to identify factors associated with suicidal ideation. An Adjusted Odds Ratio (AOR) with 95% Confidence Interval (CI) was computed to see the strength of association between outcome and independent variables. Characteristics having less than 0.05 *p*-value had been taken as significant factors associated with the outcome of interest.

**Result:**

The prevalence of suicidal ideation was found to be 8.2% (95% CI; 5.7 to 11.3) and with a standard error of 0.013. Perinatal depression (AOR=4.40, 95%CI: 1.63 11.85), not disclosed HIV status (AOR=3.73, 95%CI: 1.44 9.65), and unplanned pregnancy (AOR=2.75, 95%CI: 1.21 6.21) were significant factors associated with suicidal ideation.

**Conclusion:**

The magnitude of suicidal ideation among HIV positive perinatal women was found to be low. Perinatal depression, non-disclosed HIV status, and unplanned pregnancy were factors significantly associated with suicidal ideation. This finding suggests the integration of mental health services with maternal and HIV support programs.

**Supplementary Information:**

The online version contains supplementary material available at 10.1186/s12884-020-03529-z.

## Background

Over the past three decades, the human immunodeficiency virus (HIV) has been a global health threat [1, 2]. There are 36.9 million people living with HIV/AIDS worldwide, of which 25.7 million people live in African countries and about 85% of patients have received Highly Antiretroviral Therapy (HAART) [3]. In addition, 1.8 million children, 18.2 million were women over 15 years of age, and 940, 000 HIV/AIDS-related deaths occurred, according to the UNAIDS Global 2018 report [3, 4]. According to the UNAIDS survey, approximately 610,000 people were living with HIV/AIDS in Ethiopia and 16,000 new infections occurred in 2017, and only 59% of pregnant women with HIV/AIDS had access to antiretroviral drugs [2, 5]. Also, it was also characterized by a mixed outbreak with substantial regional heterogeneity ranging from 6.4% in the Gambela region to 0.7% in southern Ethiopia and residence (3.8% urban versus 0.6% rural) [2].

The vertical HIV transmission is one of the challenges of 2030 recognizing HIV/AIDS-free generation through the implementation of prevention of mother to child transmission strategies [2, 6, 7]. According to different evidence, during the time of perinatology, 15–20% of HIV transmission occurred from mother to fetus [8]. Diseases HIV/AIDS is the leading cause of morbidity and mortality across various population groups in developing countries. Meanwhile, HIV-related morbidities and opportunistic infections and the stigmatizing nature of the disease associated with many psychiatric illnesses have affected pregnant women.

Mental well-being is about improving the competencies of people and groups and empowering them to accomplish their self-determined objectives [9]. Suicidal ideation is a mental health disorder symptom characterized by thinking or considering, planning, and thoughts to end one’s own life. It is an all-too-common and tragic public health crisis, often done in response to overwhelming, unbearable emotional pain. Moreover, suicide is especially tragic as it is a preventable death and leaves behind many loved ones and family members [9–11].

Suicidal activity has been linked with diseases such as HIV/AIDS [12]. Suicidal activities thus span a spectrum or continuum of suicidal ideation or thought acts across a sequence of actions generally referred to as suicidal attempts or intentional self-harm [11–13]. Disease-related morbidities and increased psychosocial and mental health problems have impacted pregnant women who are HIV positive. Psychiatric conditions, including suicidal thoughts, affect the quality of life of the patient, resulting in non-adherence to their medication and treatment for HIV. This can affect their adherence to care and treatment, leading to rapid multiplication and mutation, resulting in failure of treatment [11, 14, 15].

Increased incidence of psychiatric illnesses such as major depressive disorder (MDD), suicidal thoughts, and suicidal attempts is correlated with the stigmatizing existence of HIV/AIDS [16]. A cross-sectional analysis conducted among perinatal HIV-positive women found that the prevalence of depression and ideation of suicide was 47 and 27.5% respectively [11]. Another research in South Africa (Mpumalanga) found that during pregnancy, 39% of HIV-positive pregnant women reported suicidal ideation [17]. According to previous research, major depression, partner disclosure status, stigma, age, and partner aggression have been risk factors for suicidal ideation [12, 16, 18–22]. A common problem is mental health issues, including suicidal thoughts among HIV-positive perinatal women, which affect the quality of life of the patient and feto-maternal outcomes [19]. There is however a lack of knowledge in Ethiopia regarding the severity and risk factors of suicidal ideation among perinatal women.

This study, therefore, aimed to determine the magnitude of suicidal ideations in Gondar town among HIV-positive perinatal women. The results of this study can be used as a cornerstone in the creation of effective mental health condition management guidelines, plans, and intervention services, especially suicidal ideation and attempts in PMTCT care settings. It can also provide HIV treatment professionals with clinical suggestions. For those researchers to conduct a study using a good study design, it could be used as baseline knowledge.

## Methods

### Study design, period, and setting

An institution-based cross-sectional study design was conducted among HIV positive perinatal women who had to follow up in Gondar town health institutions from February to June 2019. The study setting Gondar town is located about 748 km Northwest of Addis Ababa, Ethiopia’s capital, and has eight health centers, one university specialized hospital providing health care to the communities, including HIV care. According to the 2017 Ethiopian census report, Gondar has a total population of 358,257, and more than half of them were females. Apart from other health care services, the health facilities provide HIV chronic care, including PMTCT services for clients since 2005 as one component of comprehensive HIV/AIDS care and support programs.

### Population and samples

All HIV positive perinatal women who had a follow-up to the prevention of mother to child transmission of HIV (PMTCT) care in the selected health institutions during the data collection period was the study population. Those mothers who had at least one time visit to the follow-up of PMTCT services from the chosen settings were included. The sample size calculated using the single population proportion formula by considering the following assumptions: (p) the proportion of suicidal ideation was not done in Ethiopia (taking *p*=0.5), Z *α*/2 was taken 95% confidence level and margin of error 5%.

During the study period, there were 506 perinatal women on PMTCT follow-up and 422 women selected from eight health centers systematically. In the town, one comprehensive specialized hospital and eight public health centers provide HIV care and treatment including PMTCT services. The sample size was allocated proportionally to each of the health facilities according to the number of perinatal women on PMTCT follow-up. Thus, by using the systematic random sampling technique the final study participants were selected from those women on follow-up during data collection time.

### Data collection process

Before the actual data collection process, four data collectors and two supervisors of nurses and public health officers were recruited and one-day training was given about the objective of the study and how to interview study participants. The questionnaire initially develop in English after reviewing relevant literature and translated into the local language (Amharic) and retranslated back to English to ensure its consistency.

Data were collected by a pretested an interviewer-administered structured questionnaire for socio-demographic and mental health-related responses. Besides, clinical data such as HIV diagnosis, baseline WHO clinical stage, and a CD4 count of the clients harvested from patient medical records. The Composite International diagnostic interview (CIDI), which is adopted by the world mental health survey initiative version of WHO used to assess suicidal ideation among HIV positive perinatal women. Moreover, to measure perinatal depression we used ten items “Edinburgh Postnatal Depression Scale (EPDS)”, which has a 3 point Liker’s scale rating of the frequency of feelings ranged from never (0-point) to most of the time (3-point). To assess perceived stigma and social support, the “Oslo 3 social support scale” and “HIV stigma scales” tools were used.

In this study, suicidal ideation was the response variable. Whereas, socioeconomic factors (age, occupation, marital status, income, level of education, housing condition, residence, partner educational status). Clinical condition (clinical stage of HIV/AIDS, duration on ART, recent CD4, ART regimen, Partner HIV and disclosure status, level of hemoglobin, comorbidities), behavioral and psychosocial factors: (alcohol use, smoking, chat chewing, depression, social support, and stigma). Reproductive characteristics: (history of abortion, HIV statuses of your child, living condition) were independent variables of the study.

### The operational definitio2n of the variables

- Suicidal ideation: also known as suicidal thoughts, is thinking about, considering, or planning suicide. The range of suicidal ideation varies from fleeting thoughts to extensive thoughts, to detailed planning Suicidal attempt: suicide attempt is an attempt to try to die by suicide but survives [13].
- **Perinatal Depression:** will be measured by using the 10 items Edinburgh Postnatal Depression Scale (EPDS). In this study, if the total score is ≥ 12 for postnatal women and ≥7 for prenatal women will be categorized as having depression [23].
- Good drug adherent if the average adherence was equal to or greater than (95% or < 3 doses per month), fair drug adherent (85–94% or 4–8 doses missed per month, and poor drug adherent (less than 85% or > 9 doses missed per month) [14].
- Stigma: Participants who score above the mean score were stigmatized by measuring the HIV stigma scale
- No stigma: Participants who score below the mean score were non-stigmatized by measuring the HIV stigma scale [9]
- The Oslo Social Support (OSS-3) scores ranged from 3 to 14 with a score of 3–8 = poor support; 9–11 = moderate support; and 12–14 = strong support [14].

### Data processing and analysis

Data clean up, and cross-checking was done before analysis. After manual, cross-check, and clean up the data were coded and entered to EPI INFO version 7 then exported to STATA version 14 for analysis. Frequencies, proportion, and median with interquartile range (IQR) were used to summarize socio-demographic and clinical characteristics of study participants and presented in the form of tables, graphs, and texts. The binary logistic regression model was used to identify factors associated with perinatal depression among HIV positive perinatal women. All explanatory variables with *p*-value < 0.2 from the bivariable logistic regression model entered into the multivariable logistic regression model to control the effects of possible confounding factors. Finally, the variables with an independent association with suicidal ideation were identified based on the AOR with 95% CI and *p*-value of less than 0.05 in the final model. Hoshmer-Lemeshow goodness of fit test was done had a p-value of 0.156 and the post-estimation analysis result showed that McFadden’s pseudo-R^2^ was 0.238 and deviance of 179.034.

## Result

### Socio-demographic characteristics of the sample

A total of 414 HIV-positive pregnant women were included in the study with a response rate of 98.1%. The median age of perinatal women was 30 (IQR: 27–33) of which, 59.6% of them aged between 26 and 33 years, most (90.3%) of the women were urban dwellers, 32.1% attended secondary education, and 72.46% were married. About 37% of women had at least one child, the median number of pregnancies was 2 with (IQR: 2 to 3), and nearly one-fourth (23.6%) of women were primigravida. About 22.5% of women had a history of abortion, and 30. 4% history of child deaths. Thus, from the total participants, about 26.1% were pregnant other on follow-up and the remaining were on postnatal follow up of PMTCT services like ART drug refills, clinical evaluations, and laboratory tests of viral load measurements and dried blood sample (DBS) test of infants (Table 1).

**Table 1:** Sample description in Gondar town, Northwest Ethiopia, 2019 (*N* = 414)

Characteristics	Category	Frequency, n(%)
Age in years	18–24	66(15.9)
	26–33	247(59.7)
	≥34	101(24.4)
Residence	Urban	374(90.3)
	Rural	40(9.7)
Occupation	Had no occupation	7 (1.7)
	Government employment	97(23.4)
	Housewife	210(50.8)
	Daily laborer	59(14.3)
	Merchant	41(9.9)
Level of education	No formal education	87(21)
	Primary school	94(22.8)
	Secondary school	133(32.1)
	Diploma and above	100(24.1)
Marital status	Single	18(4.3)
	Married	300(72.5)
	Separated/Divorced	89(21.5)
	Widowed	7(1.7)
Household income in ETB	Below 1650	146 (35.3)
	1651–5195	190(45.9)
	Above 5195	78(18.8)
Living condition	Alone	94(22.7)
	With family	301(72.7)
	With husband	19(4.6)
Housing condition	Live in rented home	276(66.7)
	Own home	138(33.3)
Alcohol use	Yes	68(16.4)
	No	346(83.6)
Khat chewing	Yes	5(1.2)
	No	409(98.8)

### Clinical conditions of a perinatal women

About two-thirds (63.3%) of mothers knew their HIV status before they got pregnant, the median duration of the disease was 66 months with (IQR: 36 to 108), the majority (92.3%) of mothers had WHO stage 1, and about 63.3% women initiated antiretroviral therapy before they conceived pregnancy. Most (92.5%) of the HIV-positive perinatal women were on first-line antiretroviral therapies, of whom, about 56 and 30.7% of women were on TDF/3TC/EFV and AZT/3TC/NVP combinations, and the majority(82.4%) of them had a good level of adherence to ART medications. Most (88.7%) of the perinatal women disclosed their HIV status to at least one family member of a treatment supporter (Table 2).

**Table 2:** Clinical characteristics of HIV positive perinatal women in Gondar town health institutions, Northwest Ethiopia, 2019 (*N* = 414)

Characteristics	Category	Frequency, n (%)
When do you know your HIV status	Before pregnancy	272 (65.7)
	After pregnancy	134(32.3)
	Labor and delivery	1(0.2)
	Post-partum period	7(1.8)
Duration of HIV illness in months	≤24	100(24.2)
	25–48	62(15)
	49–72	77(18.6)
	≥73	175(42.3)
WHO stage	Stage 1	382(92.3)
	Stage 2	32(7.7)
ART regimen	First line	383(92.5)
	Second line	31(7.5)
ART regimen taking	TDF/3TC/EFV	232(56)
	AZT/3TC/NVP	127(30.7)
	TDF/3TC/NVP	20(4.8)
	TDF/3TC/ATV/r	25(6)
	AZT/3TC/EFV	8(2)
	Other	2(0.5)
Partner HIV status	Positive	246(59.4)
	Negative	62(15)
	Unknown	106(25.6)
Disclosed HIV status	Yes	367(88.6)
	No	47(11.4)
Comorbidities	Yes	130(31.4)
	No	284(68.6)
Type of comorbidities	Diabetes mellitus	4(3)
	Hypertension	2(1.5)
	Tuberculosis	124(95.5)
CD4 count	< 200	56(13.5)
	≥200	358(86.5)
Hemoglobin	< 12 mg/dl	178(43)
	≥12 mg/dl	236(57)
Syphilis test result	Negative	387(93.5)
	Positive	27(6.5)
Perinatal depression	Yes	159(38.4)
	No	255(61.6)

*TDF* Tenofovir, *3TC* Lamivudine, *EFV* Enfavirenz, *NVP* Neverapin, *AZT* Zidovudine

### Prevalence of suicidal ideation and other psychosocial problems

**Fig. 1:**
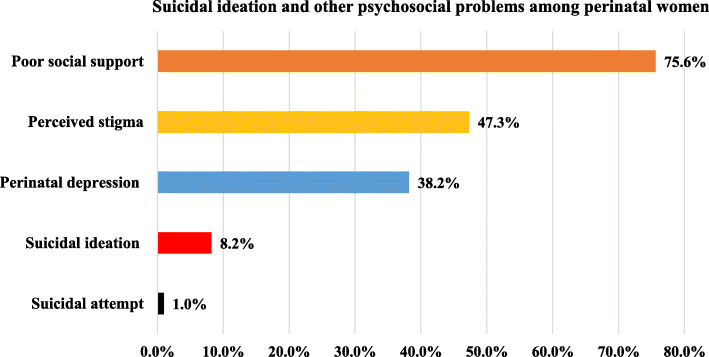
Prevalence of suicidal ideation and other psychosocial problems HIV positive perinatal women in Gondar town health (*N* = 414)

This study showed that the prevalence of suicidal ideation was 8.2% (95% CI; 5.7 to 11.3) and with the standard error of 0.013, of whom, four (0.97%) women had attempted to die by suicide, of which, poison and chemical were the used materials. Regarding the residence, suicidal ideation among rural and urban residents was 20 and 6.9%. The prevalence of suicidal though among perinatal women on ANC and PNC follow-up was 7.4 and 8.5%, and no statistically significant difference was observed (*P*-Value =0.72). The calculated mean (Standard deviation) scores of stigma and social support were 35.33 (±9.48) and 7.67 (±1.27), respectively. Moreover, this study showed that 38.4 47.3, and 75.6% had perinatal depression, perceived stigma, and poor social support, respectively (Fig. 1).

### Factors associated with suicidal ideation of HIV positive perinatal women

In the multivariable logistic regression analysis, perinatal depression, unplanned pregnancy, and non-disclosed HIV status were factors associated with suicidal ideation at a *p*-value of 0.05. For those perinatal women who had an unplanned pregnancy, the odds of suicidal ideation were 2.75 times higher than those who planned to have the pregnancy (AOR=2.75, 95%CI: 1.21 6.21). For perinatal women who did not disclose their HIV status, the odds of suicidal ideation were 3.73 times higher than those who disclosed their status (AOR=3.73, 95%CI: 1.44 9.65). Similarly, for women who had perinatal depression, the odds of suicidal ideation were 4.40 times higher than those who had no perinatal depression (AOR=4.40, 95%CI: 1.63 11.85) (Table 3).

**Table 3:** Binary logistic regression analysis to identify factors associated suicidal ideation among perinatal women (*N* = 414)

Characteristics	Suicidal ideation		Crude OR (95%CI)	Adjusted OR (95%CI)	***P***-value
	Yes	No			
**Age**					
18–25	5	61	1	1	
26–33	18	229	0.95(0.34 2.68)	1.33(0.42 4.26)	0.621
≥34	11	90	1.49(0.49 4.50)	2.31(0.63 8.41)	0.202
**Residence**					
Urban	26	348	1	1	
Rural	8	32	0.29(0.12 0.71)	0.88(0.26 3.02)	0.849
**Level of education**					
No formal education	13	74	1	1	
Primary	8	86	0.52(0.20 1.34)	0.59(0.18 1.91)	0.388
Secondary	8	125	0.36(0.14 0.92)	0.39(0.11 1.37)	0.144
Diploma and above	5	95	0.29(0.10 0.88)	0.33(0.08 1.31)	0.118
**Social support**					
Poor	30	283	1	1	
Moderate and above	4	97	0.38(0.13 1.13)	0.43(0.13 1.45)	0.177
**Planned pregnancy**					
No	20	80	5.35(2.59 11.07)	2.75(1.21 6.21)	0.015
Yes	14	300	1	1	
**HIV status disclosed**					
No	14	33	7.36(3.40 15.90)	3.73(1.44 9.65)	0.006
Yes	20	347	1	1	
**Perinatal depression**					
Yes	28	131	8.87(3.58 21.96)	4.40(1.63 11.85)	0.003
No	6	249	1	1	

## Discussion

This study revealed that the magnitude of suicidal ideation and attempt during the perinatal time was 8.2 and 1%. Characteristics including unplanned pregnancies, perinatal depression, and non-disclosed HIV status were factors associated with suicidal ideation among HIV positive perinatal women. In addition, this study also revealed that about 38.4 and 47.3% of HIV positive perinatal women suffered from depression and perceived stigma, respectively. These figures showed that psychosocial problems are rampantly high among HIV/AIDS patients in the study setting and suggests the importance of mental health interventions. Meanwhile, two-thirds of participants knew their HIV status before the recent pregnancies and births, reflecting high fertility behavior and needing robust PMTCT strategies.

This study’s magnitude of suicidal ideation was lower than previous findings of 20.5% at 72 h and 28.8% at the 6th week (Elevated risk of suicidal ideation) and systematic review finding of 26.6%. Furthermore, this finding was lower than the study results in South Africa, which reported 27.5 to 39% [12, 17]. The possible explanations might be due to the variation of socio-demographic characteristics, study settings, and year. Also, the high prevalence of intimate partner violence, extensive burden of HIV-related stigma, and social crisis were reported from those of studies in South African. Some of the reviews also assessed suicidal ideation at the time of HIV diagnosis, where emotion and frustration are high [13]. This study’s finding was also lower than the result among perinatal women in Cape Town, South Africa, where the magnitude of one-month suicidal ideation was 18% [11]. The potential explanations were reasons for differences in measuring instruments, sample population, and socio-cultural variations. Though the magnitude of suicidal thought in this study was lower, it has clinical importance as an entry point to mental health screening among perinatal women on PMTCT follow-up.

This research found that women who had unplanned pregnancies were correlated with higher suicidal ideation probabilities than those who planned to have a pregnancy. The study results in South Africa were consistent with this finding [12]. This could be because unplanned pregnancies have multiple effects, such as forced abortion, which may threaten the lives of mothers, job and school absenteeism, and the economic burden of raising the kids. Similarly, perinatal depression was associated with enhanced suicidal ideation activity in HIV positive women. The results were in line with previous studies [9, 11, 13, 16, 24, 25]. This may be due to a severe anxiety disorder and major depressive disorder (MDD) has a strong correlation with suicidal ideation [10]. Those patients who thought to kill themselves may have suicidal symptoms from the start, and patients who attempted to die by themselves are more depressed and nervous. Depression, however, may also have been aroused from suicidal ideation and unsuccessful attempts, which is the limitation of the nature of the cross-sectional study design, which contributes to confusion about the cause-effect relationship.

Likewise, this study also showed that perinatal women who did not disclose their HIV status associated with increased suicidal ideation than those disclosed. This could be because women who had not disclosed HIV status might have high perceived stigma and isolation. Also, that non-disclosure status affects the level of social support of the victims.

In previous studies in South Africa, intimate partner violence, depression, increased income, stigma, younger age, disclosure of HIV status to partner were significant factors associated with suicidal ideation among HIV positive perinatal women [10]. These variables, however, had no statistically significant association in our sample. This may be because of the variation in sample size and study population. The study was conducted in South Africa among women living in rural areas. Whereas, the majority (90.7%) of women in this work were urban residents where adequate HIV treatment available and accessible.

### Strength and limitation

This study has strengths in determining the magnitude of psychiatric disorders among perinatal women, which could help clinicians to intervene and be used as an entry point to perform screening for mental illnesses. This study has limitations of the cross-sectional study design, which may be linked to reverse causation. In addition, the problem’s sensitive nature may introduce social desirability bias, which could underestimate the magnitude of the problem. Furthermore, factors associated with suicidal attempts were not assessed due to the rarity of events.

## Conclusion

The magnitude of suicidal ideation among HIV positive perinatal women was found to be low. Factors substantially associated with suicidal ideation were perinatal depression, non-disclosed HIV status, and unplanned pregnancy. The alignment of mental health services with maternal and HIV support system is indicated by these findings. This study has implications for the enhancement of maternal services and HIV treatment programs for HIV positive perinatal women, clinicians, healthcare managers. Furthermore, the results of this study may help to integrate HIV treatment, maternal and mental health services at the bridge of care. In addition, in this research, the magnitude of suicidal ideation, attempt, and perinatal depression may be used as an entry point for routine mental health screening and evidence-based interventions.

## Supplementary Information

**Additional file 1.** Data collections tools used for this study.

## Data Availability

The datasets used during the current study is available from the corresponding author.

## Abbreviations

AOR: Adjusted Odds Ratio
CI: Confidence Interval
CIDI: Composite International Diagnostic Interview
ETB: Ethiopia Birr
HIV/AIDS: Human Immune Virus/ Acquired Immune Deficiency Syndrome
IQR: Interquartile Range
MDD: Major Depressive Disorder
PMTCT: Prevention of Mother to Child transmission
OSS: Oslo Social Support
SPSS: Statistical Package for Social Science
VDRL: Vernal Disease Research Laboratory
WHO: World Health Organization
