# Supplementary material for: Prevalence of suicidal ideation and associated factors among HIV positive perinatal women on follow-up at Gondar town health institutions, Northwest Ethiopia: a cross-sectional study
Source: BMC Pregnancy Childbirth. 2021 Jan 9;21:42. doi: 10.1186/s12884-020-03529-z (PMC7797091; doi:10.1186/s12884-020-03529-z)
Supplement: Supplementary file 1 — Additional file 1. Data collections tools used for this study. [file 12884_2020_3529_MOESM1_ESM.docx]

# **Supplementary file**

| 1. **Socio-demographic characteristics** | | | |
| --- | --- | --- | --- |
| **Q101** | Age | ----------- years |  |
| **Q102** | Marital status | 1. Single 2. married 3. Divorced 4. Widowed 5. separated |  |
| **Q103** | Residence | 1. Urban 2.Rural |  |
| **Q104** | Level of education | 1. unable to read and write 2. able to read and write only 3. Primary school 4. Secondary school 5. Diploma and above |  |
| **Q105** | Living status | 1. Alone 2. With family 3.with husband 4. With friends |  |
| **Q106** | Occupation | 1.unemoloyed  2.Gov. employed  3.Merchant  4.Farmer  5.House wife  6.Daily labor  7.no occupation |  |
| **Q107** | Income | ------ETB/month |  |
| **Q108** | In what types of house do you live? | 1. In a rented accommodation 2. With a renting relative 3. In own home 4. With relative owning home |  |

| 1. **Reproductive Health characteristics**   The next few questions are about any previous pregnancies and children you might have had. | | | | | | |  |
| --- | --- | --- | --- | --- | --- | --- | --- |
| **Q201** | How many times have you been pregnant, including this pregnancy? | | ---- | | |  |  |
| **Q202** | Have you experienced of child death? | | 1.Yes 2.No | | |  |  |
| **Q203** | History of abortion | | 1.Yes 2.No | | |  |  |
| **Q204** | Hx still birth | | 1.Yes 2.No | | |  |  |
| **Q205** | Was planned to be pregnant or to have this child | | 1.Yes 2.No | | |  |  |
| **Q206** | Do you have another child? | | 1. Yes 2. No | | | If yes go to **Q205** |  |
| **Q207** | How many children do you have? | | _____ | | |  |  |
| **Q208** | Are they tested to HIV? | | 1. Yes 2. no | | | If yes go to **Q209** |  |
| **Q209** | What was the result? | | 1. Yes 2. no | | |  |  |
| 1. **Behavioral factors** | | | | | | | |
| **Q301** | Are you Currently use of Alcohol? | 1/ yes 2/ no | |  |  | | |
| **Q302** | Have you currently smoking Cigarettes? | 1/ yes 2/ no | |  |  |  |  |
| **Q303** | Are you using chat, shisha, or other substances? | **1**/ yes 2/ no | |  |  |  |  |

| 1. **Part IV Social support and stigma scale *Explanation of the Oslo-3 Social Support Scale (OSS-3) (source; Dalgard et al., 2006)***  \| **Q401**. How many people are so close to you that you can count on them if you have a serious problem?  None 1  1 or 2 2  3-5 3  6 or more 4  **Q402**. How much concern do people show in what you are doing?  A lot of concern and interest 5  Some concern and interest 4  Uncertain 3  Little concern and interest 2  No concern 1  **Q403**. How easy can you get practical help from neighbors if you should need it?  Very easy 5  Easy 4  Possible 3  Difficult 2  Very difficult 1 \| \| --- \|   **4. EPDS tool for screening depression**   \| **Feelings of depression (EPDS)**. **Tell us the way you have been feeling in the past 1wk (1ek including today. In the past seven days,** \| \| \| \| \| --- \| --- \| --- \| --- \| \| **Q501** \| In the last week, have you been able to laugh and see the funny side of things? \| As much as I always used to  Not as much as I used to  Certainly not as much as I used to  Not at all \| 0  1  2  3 \| \| **Q502** \| In the last week, have you looked forward with enjoyment to things? \| As much as I always used to  Rather less  Certainly less  Never looked forward \| 0  1  2  3 \| \| **Q503** \| In the last week, have you blamed yourself unnecessarily when things went wrong? \| Most of the time  Sometimes  Rarely  Never \| 3  2  1  0 \| \| **Q504** \| In the last week, have you been anxious or worried for no good reason? \| Most of the time  Sometimes  Not often  Never \| 0  1  2  3 \| \| **Q505** \| In the last week, have you felt scared or panicky for no good reason? \| Most of the time  Sometimes  Rarely  Never \| 3  2  1  0 \| \| **Q506** \| In the last week, have things been getting on top of you? \| Most of the time unable to cope  Sometimes unable  Mostly able  Coping as usual \| 3  2  1  0 \| \| **Q507** \| In the last week, have you been so unhappy that you have had difficulty sleeping? \| Most of the time  Sometimes  Rarely  Never \| 3  2  1  0 \| \| **Q508** \| In the last week, have you felt sad or miserable? \| Most of the time  Sometimes  Occasionally  Never \| 3  2  1  0 \| \| **Q509** \| In the last week, have you felt so unhappy that you have been crying? \| Most of the time  Sometimes  Occasionally  Never \| 3  2  1  0 \| \| **Q510** \| In the last week, has the thought of harming yourself occurred to you? \| Frequently  Sometimes  Not often  Never \| 3  2  1  0 \|   **Part 5. Questions related with HIV-related stigma**  **HIV stigma scale**   \|  \| \| 1 \| 2 \| 3 \| 4 \| 5 \|  \| 5 \| \| --- \| --- \| --- \| --- \| --- \| --- \| --- \| --- \| --- \| \| Disagree strongly \| Disagree  Slightly \| Neutral \| Agree slightly \| Agree strongly \| \| **Q601** \| I work hard to keep my HIV a secret \|  \|  \|  \|  \|  \|  \|  \| \| **Q602** \| Most people believe a person who has HIV is dirty \|  \|  \|  \|  \|  \|  \|  \| \| **Q603** \| Having HIV makes me feel unclean \|  \|  \|  \|  \|  \|  \|  \| \| **Q604** \| Most people think a person with HIV is disgusting \|  \|  \|  \|  \|  \|  \|  \| \| **Q605** \| Having HIV makes me feel I'm a bad person \|  \|  \|  \|  \|  \|  \|  \| \| **Q606** \| Most with HIV are rejected when others learn \|  \|  \|  \|  \|  \|  \|  \| \| **Q607** \| I am very careful whom I tell that I have HIV \|  \|  \|  \|  \|  \|  \|  \| \| **Q608** \| Having HIV in my body feels disgusting \|  \|  \|  \|  \|  \|  \|  \| \| **Q609** \| I have been hurt by how people reacted to learning I have HIV \|  \|  \|  \|  \|  \|  \|  \| \| **Q610** \| I worry that people who know I have HIV will tell others \|  \|  \|  \|  \|  \|  \|  \| \| **Q611** \| I have stopped socializing with some due to their reactions to my HIV \|  \|  \|  \|  \|  \|  \|  \| \| **Q612** \| I have lost friends by telling them I have HIV \|  \|  \|  \|  \|  \|  \|  \| |
| --- | --- | --- | --- | --- | --- | --- | --- | --- | --- | --- | --- | --- | --- | --- | --- | --- | --- | --- | --- | --- | --- | --- | --- | --- | --- | --- | --- | --- | --- | --- | --- | --- | --- | --- | --- | --- | --- | --- | --- | --- | --- | --- | --- | --- | --- | --- | --- | --- | --- | --- | --- | --- | --- | --- | --- | --- | --- | --- | --- | --- | --- | --- | --- | --- | --- | --- | --- | --- | --- | --- | --- | --- | --- | --- | --- | --- | --- | --- | --- | --- | --- | --- | --- | --- | --- | --- | --- | --- | --- | --- | --- | --- | --- | --- | --- | --- | --- | --- | --- | --- | --- | --- | --- | --- | --- | --- | --- | --- | --- | --- | --- | --- | --- | --- | --- | --- | --- | --- | --- | --- | --- | --- | --- | --- | --- | --- | --- | --- | --- | --- | --- | --- | --- | --- | --- | --- | --- | --- | --- | --- | --- | --- | --- | --- | --- | --- | --- | --- | --- | --- | --- | --- | --- | --- | --- | --- | --- | --- | --- | --- | --- | --- | --- | --- | --- | --- | --- |

**Part 6. Questions related to Suicidal ideation and Attempt (**CIDI**)**

| Q613 | Ever thought about committing suicide | 1. Yes 2 No |
| --- | --- | --- |
| Q614 | Ever attempted to suicide | 1. Yes 2 No |

|  |
| --- |

**7. Clinical and health related factors**

| **Q701** | Comorbid illnesses (DM, HTN, TB…) | Others (specify)_______ |  |
| --- | --- | --- | --- |
| **Q702** | Hx of mental illness | 1. Yes 2.No |  |
| **Q703** | Family Hx of any mental illness | 1.Yes 2.No |  |
| **Q704** | Baseline CD4 count | ____cells/mm3 |  |
| **Q705** | Current CD4count | _____cell/mm3 |  |
| **Q706** | Most recent Viral load | _______copies/ml |  |
| **Q707** | Syphilis test result | 1. Negative 2. Positive |  |
| **Q708** | Hgb level | __gm/dl |  |
| **Q709** | Current WHO clinical staging of a women | 1.stage I 2.stage II 3.stage III  4.stage IV |  |
| **Q710** | Regimen of ART | 1.First line 2.Second line |  |
| **Q711** | Specific regimen | ______________ |  |
| **Q712** | Duration of ART | ________months/years |  |
| **Q713** | Adherence to ART | 1.Good 2.Fair 3.poor |  |
| **Q714** | When did you get tested? | 1. Before pregnancy 2. During pregnancy 3. During labor and delivery   4.During post-partum |  |
| **Q715** | When did the women started to ART? | 1. Before pregnancy 2. During pregnancy 3. During labor and delivery 4. During post-partum |  |
| **Q716** | Have you disclosed your HIV status? | 1.Yes 2.No | If yes |
| **Q717** | For whom you disclosed? | 1.Partner 2.Sister 3.Mother 4.Others(specify)_____ |  |
| **Q718** | HIV status of the partner? | 1. Positive 2. Negative 3.Unknown |  |
| **Q719** | ART status of the partner | 1. On ART 2. Not On ART |  |
